# TyG-BMI as a predictor of ischemic stroke over 10 years in middle-aged and older adults: findings from the China cardiometabolic disease and cancer cohort (4C) study

**DOI:** 10.3389/fneur.2025.1609853

**Published:** 2025-07-02

**Authors:** Ying Miao, Yu Wang, Yuting He, Pijun Yan, Qin Wan

**Affiliations:** ^1^Department of Endocrinology and Metabolism, Affiliated Hospital of Southwest Medical University, Luzhou, China; ^2^Metabolic Vascular Disease Key Laboratory of Sichuan Province, Luzhou, China; ^3^Sichuan Clinical Research Center for Diabetes and Metabolism, Luzhou, China; ^4^Sichuan Clinical Research Center for Nephropathy, Luzhou, China; ^5^Cardiovascular and Metabolic Diseases Key Laboratory of Luzhou, Luzhou, China; ^6^Department of Cardiology, Luzhou People’s Hospital, Luzhou, China; ^7^Department of Neurology, Chengdu Sixth People's Hospital, Chengdu, China

**Keywords:** triglyceride-glucose index, body mass index, TyG-BMI, ischemic stroke, insulin resistance

## Abstract

**Background:**

Ischemic stroke (IS) is a leading cause of death and disability, imposing a significant economic burden globally. Research has demonstrated that insulin resistance (IR) plays a key role in the development of atherosclerosis, platelet dysfunction, and a hypercoagulable state, all of which contribute to the pathogenesis and progression of IS. The triglyceride-glucose (TyG) index serves as a practical tool for assessing insulin sensitivity, with previous studies exploring its correlation with IS. However, the relationship between the novel TyG-body mass index (TyG-BMI), which combines TyG with body mass index (BMI) as a measure of general obesity, and IS remains unclear. Therefore, this study employs a prospective design to assess the predictive value of TyG-BMI for the 10-year risk of IS in individuals without intervention.

**Methods:**

The study population was derived from the China Cardiometabolic Disease and Cancer Cohort (4C) Study, predominantly comprising participants from Luzhou City, Sichuan Province, and primarily targeting individuals aged 40 and above. Comprehensive data collection was conducted using both questionnaires and specialized medical equipment, covering physical measurements, blood pressure, and relevant biochemical markers. Participants with a history of stroke were excluded from the study. Based on the initial data, participants were divided into four groups according to the TyG-BMI quartiles. Spearman correlation analysis was used to examine the relationship between TyG-BMI and clinical and laboratory parameters. The Log-rank test was applied to analyze differences in the cumulative incidence of IS among the four groups. The Cox proportional hazards model was used to analyze the relationship between TyG-BMI and the 10-year incidence of new IS. Additionally, the ROC curve was employed to assess the predictive value of TyG-BMI for the 10-year incidence of new IS in the middle-aged and elderly population.

**Results:**

This study included 9,406 participants, consisting of 3,139 males (33.4%) and 6,267 females (66.6%). During the non-interventional follow-up period of 10 years, 483 deaths were recorded, resulting in a mortality rate of 5.1%. In addition, 527 new cases of IS were reported, yielding an incidence rate of 5.6%. The Log-rank test revealed a significant increase in the cumulative incidence of IS across increasing TyG-BMI quartiles (*p* < 0.01). Furthermore, Cox regression analysis identified a significant correlation between TyG-BMI levels, as a risk factor, and the occurrence of IS. After adjusting for other risk factors, the risk of developing new IS in the Q2 group was 1.449 times that of the Q1 group (*p* = 0.012), while the risk in the Q3 group was 1.438 times that of the Q1 group (*p* = 0.014), and the risk in the Q4 group was 1.434 times that of the Q1 group (*p* = 0.020). ROC curve analysis showed that, in the overall study population, TyG-BMI demonstrated a predictive value for new IS over 10 years (AUC = 0.566, 95% CI = 0.542–0.590, *p* < 0.001), with a cutoff value of 204.1307, sensitivity of 64.3%, and specificity of 47.8%. In male participants, TyG-BMI showed a predictive value for new IS over 10 years (AUC = 0.537, 95% CI = 0.501–0.574, *p* = 0.067), with a cutoff value of 195.1996, sensitivity of 73.8%, and specificity of 37.0%. In female participants, TyG-BMI demonstrated a predictive value for new IS over 10 years (AUC = 0.583, 95% CI = 0.551–0.615, *p* < 0.001), with a cutoff value of 204.295, sensitivity of 65.8%, and specificity of 48.7%.

**Conclusion:**

This study revealed a significant association between TyG-BMI and the 10-year incidence of new-onset IS among middle-aged and elderly individuals, indicating that TyG-BMI may serve as a valuable predictive marker for assessing IS risk in this population.

## Introduction

Ischemic stroke (IS) is an acute cerebrovascular disorder resulting from insufficient blood supply to the brain, which is characterized by high rates of morbidity, disability, recurrence, and mortality (1). The Global Burden of Disease study has shown that the mortality associated with stroke and its complications has been increasing annually. Since 2015, stroke has become the leading cause of death and disability among middle-aged and elderly individuals in China (2, 3). According to the 2020 Seventh National Population Census of China, approximately 17.8 million people aged 40 and older have experienced a stroke, with an estimated 3.4 million new cases and 2.3 million stroke-related deaths (4). With shifts in diet and lifestyle, the incidence of stroke has been rising, and the disease is increasingly affecting younger populations. While advances in emergency medical care have lowered stroke mortality rates, the resulting neurovascular damage often leads to persistent sequelae such as swallowing difficulties and motor impairments, which significantly reduce the quality of life for patients and impose an additional burden on families and society. Consequently, early identification of high-risk groups for stroke has become more critical than ever (5, 6).

Obesity is a significant global public health issue. Studies have identified obesity as a modifiable risk factor for stroke (7) and have shown that it is strongly associated with an increased risk of atherosclerotic vascular disease (8). Some research has indicated that, after adjusting for known cardiovascular risk factors, body mass index (BMI) is not significantly correlated with stroke risk (8, 9). However, other studies suggest that BMI is an effective predictor of cardiovascular diseases (10).

Insulin resistance (IR) is recognized as one of the significant risk factors for IS. Numerous biomarkers are available for assessing IR, each varying in terms of technical sensitivity, limitations, and complexity. Currently, there are several methods and biomarkers used to evaluate IR in both clinical and research settings, and they differ in sensitivity, limitations, and complexity. The hyperinsulinemic-euglycemic clamp (HIEC) is widely considered the gold standard for assessing IR due to its high accuracy and sensitivity. However, its application in large-scale clinical practice is limited by its technical complexity, invasiveness, and high cost (11). Other indicators based on serum insulin levels, such as the homeostasis model assessment for insulin resistance (HOMA-IR), the oral glucose insulin sensitivity index, and the McAuley index (11, 12), are not suitable for diabetic patients undergoing insulin treatment. Additionally, the difficulty of performing insulin assays in laboratory settings in underdeveloped areas limits the applicability of these indicators in large-scale epidemiological studies. The triglyceride-glucose (TyG) index has been proposed as a simple and reliable alternative for assessing IR (13). Recently, several TyG-derived indices, calculated by multiplying the TyG index with anthropometric measures, have been introduced, and their predictive value for cardiovascular disease (CVD) risk has been explored (14). Stroke, a major type of CVD, is associated with the TyG index, though some study results remain inconsistent.

This study employs a prospective cohort design to investigate the predictive value and optimal cutoff points of the TyG-BMI index for assessing the risk of IS, with the goal of providing scientific evidence for the early prevention and management of IS.

## Methods

### Study population

The study population was drawn from the China Cardiometabolic Disease and Cancer Cohort (4C) Study, primarily consisting of participants from Luzhou City, Sichuan Province. Between April and November 2011, we recruited 10,150 individuals, all aged 40 years or older. The inclusion criteria were as follows: (1) Residing in the area for at least 5 years; (2) Voluntary participation in follow-up; (3) Age 40 years or older. The exclusion criteria included: (1) History of IS; (2) Age under 40 years; (3) Limited mobility; (4) Refusal to participate in follow-up; (5) Incomplete data. Following thorough screening, 9,406 individuals met the criteria and were included in the study cohort.

### Baseline survey

A baseline survey was conducted in 2011, which included the following components: (1) Trained researchers conducted face-to-face interviews to gather information on participants’ gender, age, medical history (including hypertension, diabetes, and stroke), family history of diabetes, and smoking history.(2) Participants’ weight and height were measured before breakfast, followed by the calculation of body mass index (BMI). Seated systolic blood pressure (SBP) and diastolic blood pressure (DBP) were measured every 5 min, with three readings taken. The average of these readings was then calculated. (3) Participants fasted for at least 10 h before undergoing a 75-gram oral glucose tolerance test. Blood samples were collected at baseline (0 h) and 2 h after the test, and stored at −80°C. Blood glucose measurements included glycated hemoglobin A1c (HbA1c), fasting blood glucose (FBG), and 2-h postprandial blood glucose (PBG), all measured using the glucose oxidase method. The lipid profile was assessed by measuring low-density lipoprotein cholesterol (LDL-C), high-density lipoprotein cholesterol (HDL-C), total cholesterol (TC), and triglycerides (TG). Biochemical tests included alanine aminotransferase (ALT), aspartate aminotransferase (AST), gamma-glutamyl transferase (GGT), and creatinine (Crea) levels. All tests were conducted in accordance with the International Organization for Standardization (ISO) 15,189 guidelines in a certified central laboratory (15).

### Follow-up survey

A five-year follow-up was conducted in 2016, comprising face-to-face visits, physical examinations, and laboratory tests. For participants who were unable to attend in-person assessments, follow-up was carried out via telephone. In 2021, a 10-year follow-up of the same study population was performed, during which data on chronic disease and mortality were obtained from the Luzhou Municipal Health Bureau and the Luzhou Center for Disease Control and Prevention (15).

### Definitions

TyG and TyG-BMI were calculated using the following formulas: (1) BMI = body mass (kg) /height^2^ (m^2^) (15); (2) TyG = ln [triglycerides (mg/dL) × glucose (mg/dL) /2] (13); (3) TyG-BMI = TyG × BMI (15).

### Statistical analysis

The primary characteristics of the sample were presented using descriptive statistics. Continuous variables were described as either the mean ± standard deviation (SD) or the median (interquartile range, IQR), depending on their distribution. Categorical variables were represented as counts (percentages). Comparisons of continuous variables were conducted using the Student’s t-test, Mann–Whitney U test, Kruskal-Wallis H test, or one-way ANOVA, depending on data normality. Chi-square tests were used for comparisons of categorical variables between groups. Pairwise comparisons among multiple groups were performed using the Bonferroni method. Cumulative incidence was calculated using the Kaplan–Meier method, and the log-rank test was used to compare the cumulative incidence across four groups based on TyG-BMI quartiles. A Cox proportional hazards model was further applied to assess the relationship between TyG-BMI and the 10-year incidence of new IS during non-intervention follow-up. In all statistical analyses, *p*-values were two-tailed, with significance set at *p* < 0.05. All analyses were performed using SPSS software (version 26.0).

## Results

### Baseline characteristics

The study enrolled 9,406 participants, including 3,139 men (33.4%) and 6,267 women (66.6%). During the 10-year non-interventional follow-up period, 483 participants died from various causes, resulting in a mortality rate of 5.1%, while 527 individuals developed new-onset IS, yielding an incidence rate of 5.6%. Baseline characteristics stratified by TyG-BMI quartiles are presented in Table 1. From the lowest (Q1) to the highest (Q4) quartile, there was a gradual increase in age, as well as in the prevalence of prediabetes, diabetes, and hypertension. Similarly, levels of systolic SBP, DBP, body weight, BMI, TG, TC, FPG, 2hPG, HbA1c, ALT, AST, GGT, and creatinine showed significant upward trends (all *p* < 0.05). Conversely, the proportion of participants with normal glucose metabolism and levels of HDL-C decreased progressively across the quartiles (*p* < 0.05). Additionally, the incidence of IS, pulse rate, height, and LDL-C varied significantly among the four groups (*p* < 0.05). In contrast, no significant differences were observed across quartiles in terms of sex distribution, prevalence of myocardial infarction, family history of diabetes, smoking habits, or alcohol consumption (all *p* > 0.05).

**Table 1:** Baseline characteristics of study subjects according to TyG-BMI categories.

Variables	All	Q1 (*n* = 2,352) (112.5000–182.7250)	Q2 (*n* = 2,352) (182.7250–207.1100)	Q3 (*n* = 2,351) (207.1100–233.0306)	Q4 (*n* = 2,351) (233.0306–391.3500)	*p*-value
Gender (%)						0.167
Male	3,139 (33.40%)	761 (32.40%)	758 (32.20%)	817 (34.70%)	803 (34.20%)	
Female	6,267 (66.60%)	1,590 (67.60%)	1,594 (67.80%)	1,535 (65.30%)	1,548 (65.80%)	
Age (years)	58.00 (51.00,65.00)	56.00 (48.00,64.00)^bcd^	58.00 (50.00,65.00)^acd^	59.00 (53.00,66.00)^ab^	60.00 (53.00,66.00)^ab^	<0.001
Glycemic status						<0.001
Normal	5,061 (53.80%)	1731 (73.60%)^bcd^	1,425 (60.60%)^acd^	1,076 (45.70%)^abd^	829 (35.30%)^abc^	
Prediabetes	2,636 (28.00%)	495 (21.10%)^bcd^	664 (28.20%)^ac^	769 (32.70%)^ab^	708 (30.10%)^a^	
Diabetes	1709 (18.20%)	125 (5.30%)^bcd^	263 (11.20%)^acd^	507 (21.60%)^abd^	814 (34.60%)^abc^	
Myocardial infarction	35 (0.40%)	6 (0.30%)	7 (0.30%)	9 (0.40%)	13 (0.60%)	0.388
Coronary heart disease	323 (3.50%)	70 (3.10%)	66 (2.90%)^d^	84 (3.70%)	103 (4.50%)^b^	0.012
Hypertension (%)	1,571 (17.20%)	301 (13.10%)^cd^	359 (15.70%)^d^	410 (17.90%)^ad^	501 (22.00%)^abc^	<0.001
Family History of Diabetes (%)	902 (9.60%)	202 (8.60%)	251 (10.70%)	218 (9.30%)	231 (9.80%)	0.095
Smoking (%)	1,331 (15.40%)	355 (16.40%)	327 (15.20%)	334 (15.50%)	315 (14.40%)	0.359
Alcohol consumption (%)	2,563 (29.20%)	626 (28.70%)	663 (30.30%)	673 (30.50%)	601 (27.40%)	0.073
SBP (mmHg)	125.00 (111.00,141.00)	115.00 (104.00,129.00)^bcd^	123.00 (110.00,138.00)^acd^	128.00 (116.00,143.00)^abd^	134.00 (120.00,148.00)^abc^	<0.001
DBP (mmHg)	77.00 (70.00,84.00)	72.00 (66.00,80.00)^bcd^	76.00 (69.00,83.00)^acd^	78.00 (71.00,86.00)^abd^	80.00 (74.00,89.00)^abc^	<0.001
Pulse (beats per minute)	79.00 (72.00,87.00)	79.00 (72.00,86.00)^d^	79.00 (72.00,87.00)^d^	79.00 (72.00,86.00)^d^	80.00 (74.00,88.00)^abc^	<0.001
Height (cm)	157.00 (152.00,162.50)	158.00 (153.00,162.50)^d^	157.00 (152.00,162.30)	157.00 (152.00,163.00)	156.20 (151.50,163.00)^a^	<0.001
Weight (Kg)	58.50 (52.50,65.50)	50.00 (46.00,54.50)^bcd^	56.50 (52.28,61.00)^acd^	61.35 (57.00,66.00)^abd^	68.00 (62.30,74.50)^abc^	<0.001
BMI	23.70 (21.60,26.00)	20.30 (19.10,21.20)^bcd^	22.90 (21.90,23.70)^acd^	24.80 (23.90,25.80)^abd^	27.60 (26.20,29.30)^abc^	<0.001
TG (mmol/l)	1.28 (0.90,1.88)	0.86 (0.68,1.11)^bcd^	1.15 (0.88,1.50)^acd^	1.48 (1.10,1.98)^abd^	2.05 (1.48,3.03)^abc^	<0.001
TC (mmol/l)	4.56 (3.81,5.32)	4.25 (3.51,5.00)^bcd^	4.51 (3.77,5.23)^acd^	4.66 (3.92,5.44)^abd^	4.80 (4.11,5.55)^abc^	<0.001
HDL-C (mmol/l)	1.22 (1.00,1.45)	1.35 (1.11,1.62)^bcd^	1.27 (1.05,1.49)^acd^	1.19 (0.99,1.40)^abd^	1.09 (0.92,1.28)^abc^	<0.001
LDL-C (mmol/l)	2.52 (1.99,3.10)	2.29 (1.77,2.82)^bcd^	2.54 (2.01,3.11)^acd^	2.66 (2.11,3.24)^ab^	2.63 (2.11,3.21)^ab^	<0.001
FPG (mmol/l)	5.45 (5.11,6.02)	5.20 (4.93,5.52)^bcd^	5.36 (5.07,5.78)^acd^	5.57 (5.22,6.15)^abd^	5.85 (5.38,6.93)^abc^	<0.001
2hPG (mmol/l)	7.81 (6.39,10.45)	6.73 (5.74,8.14)^bcd^	7.31 (6.19,9.24)^acd^	8.41 (6.82,11.27)^abd^	9.70 (7.54,13.22)^abc^	<0.001
HbA1c (%)	5.90 (5.60,6.30)	5.80 (5.50,6.10)^bcd^	5.80 (5.60,6.20)^acd^	6.00 (5.70,6.40)^abd^	6.20 (5.80,6.80)^abc^	<0.001
ALT (U/L)	12.00 (9.00,18.00)	10.00 (7.00,14.00)^bcd^	11.00 (8.00,16.00)^acd^	13.00 (9.00,19.00)^abd^	16.00 (11.00,24.00)^abc^	<0.001
AST (U/L)	19.00 (16.00,24.00)	18.00 (15.00,23.00)^cd^	19.00 (15.00,23.00)^cd^	20.00 (16.00,24.00)^abd^	21.00 (17.00,27.00)^abc^	<0.001
GGT (U/L)	18.00 (13.00,29.00)	14.00 (10.00,20.00)^bcd^	16.00 (12.00,24.00)^acd^	20.00 (14.00,31.00)^abd^	25.00 (17.00,41.00)^abc^	<0.001
Crea (umol/l)	62.40 (55.40,70.60)	60.10 (53.10,67.60)^bcd^	61.60 (54.90,69.90)^acd^	63.40 (56.50,71.70)^abd^	64.40 (57.20,73.40)^abc^	<0.001
TyG	8.66 (8.28,9.11)	8.19 (7.93,8.46)^bcd^	8.53 (8.25,8.80)^acd^	8.82 (8.53,9.16)^abd^	9.24 (8.87,9.68)^abc^	<0.001

Q1, First quartile group; Q2, Second quartile group; Q3, Third quartile group; Q4, Fourth quartile group. Superscripts indicate statistically significant differences: “a” denotes a significant difference compared to the Q1 group; “b” denotes a significant difference compared to the Q2 group; “c” denotes a significant difference compared to the Q3 group; “c” denotes a significant difference compared to the Q3 group; “d” denotes a significant difference compared to the Q4 group.

### TyG-BMI and its association with clinical and laboratory variables

Spearman correlation analysis was performed with TyG-BMI as a continuous variable. As shown in Table 2, TyG-BMI was significantly and positively correlated with a range of variables, including age, male sex, coronary heart disease, hypertension, SBP, DBP, pulse rate, body weight, BMI, TG, TC, LDL-C, FPG, 2hPG, HbA1c, ALT, AST, GGT, crea, and the TyG index (all *p* < 0.05). In contrast, TyG-BMI showed significant negative correlations with height and HDL-C (both *p* < 0.05).

**Table 2:** TyG-BMI and its association with clinical and laboratory variables.

Indicators	*r*	*p* value
Age	0.111**	<0.001
Gender (female vs. male)	−0.016	0.11
Glycemic status	0.241**	<0.001
Myocardial infarction (yes vs. no)	−0.02	0.06
Coronary heart disease (yes vs. no)	0.032**	0.002
Hypertension (yes vs. no)	0.090**	<0.001
Family history of diabetes	0.012	0.249
Smoking (yes vs. no)	−0.018	0.086
Drinking (yes vs. no)	−0.009	0.407
SBP	0.320**	<0.001
DBP	0.285**	<0.001
Pulse	0.066**	<0.001
Height	−0.035**	0.001
Weight	0.731**	<0.001
BMI	0.916**	<0.001
TG	0.634**	<0.001
TC	0.195**	<0.001
HDL-C	−0.278**	<0.001
LDL-C	0.171**	<0.001
FPG	0.388**	<0.001
2hPG	0.380**	<0.001
HbA1c	0.278**	<0.001
ALT	0.337**	<0.001
AST	0.175**	<0.001
GGT	0.363**	<0.001
Crea	0.158**	<0.001
TyG	0.671**	<0.001

* represents *p*<0.05; ** represents *p*<0.01.

### Log-rank test

**Figure 1:**
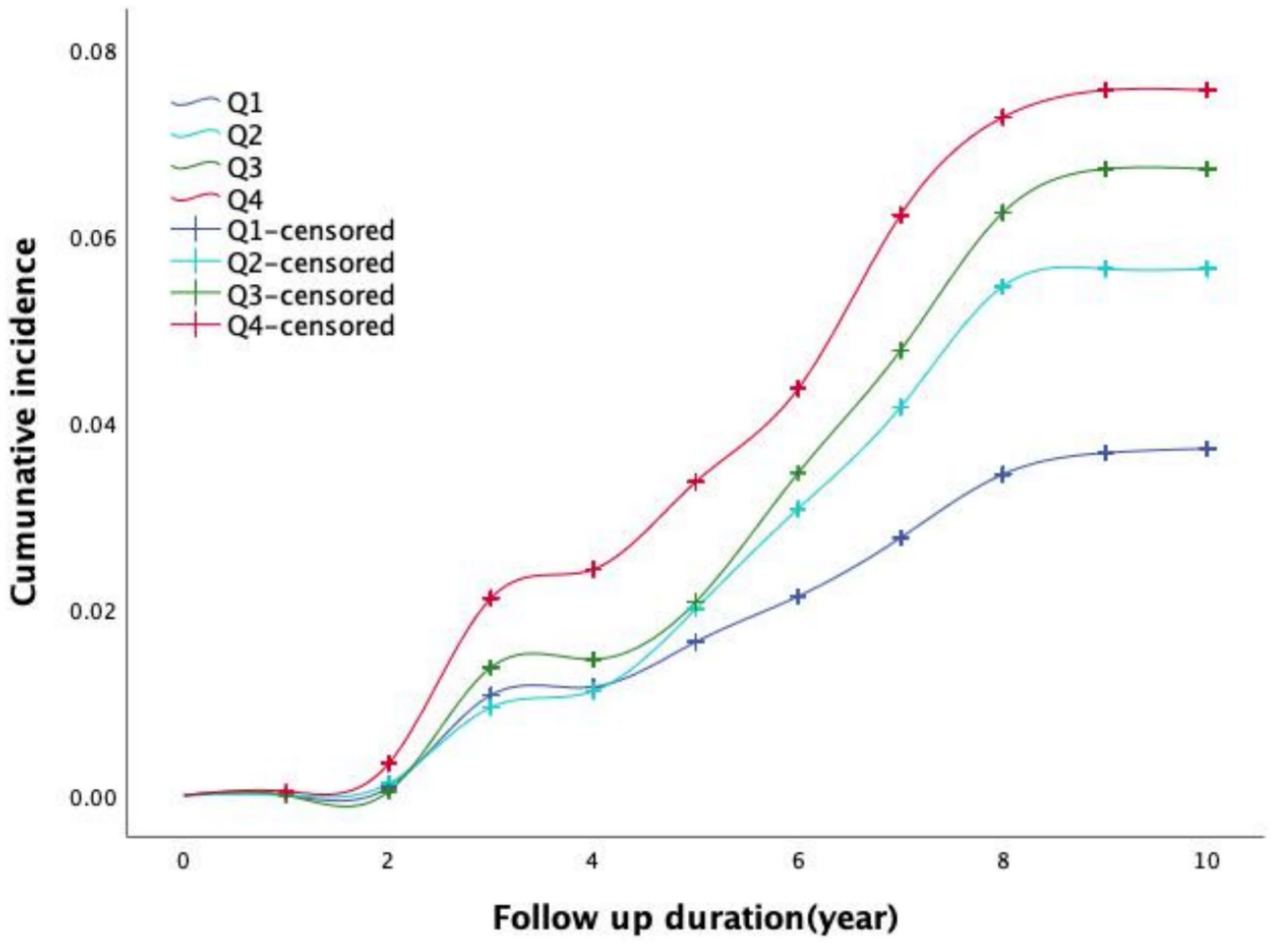
Cumulative incidence of ischemic stroke in different baseline TyG-BMI categories.

Figure 1 illustrates the time-dependent trends in the cumulative incidence of IS across different TyG-BMI quartile groups. The log-rank test (χ^2^ = 31.545, *p* < 0.001) revealed significant differences in the cumulative incidence curves among the four groups. Participants in higher TyG-BMI quartiles exhibited a greater cumulative incidence of IS. Moreover, the disparity in incidence rates among the groups became increasingly pronounced over the follow-up period.

### Univariate analysis of determinants of IS

Table 3 presents the associations between TyG-BMI and other variables with the risk of developing IS. The univariate analysis identified significant associations between IS and several factors, including gender, age, glycemic status, myocardial infarction, coronary heart disease, hypertension, family history of diabetes, weight, BMI, pulse, TG, HDL-C, ALT, AST, Cre, TyG, and TyG-BMI.

**Table 3:** Univariate analysis of risk factors for ischemic stroke in study subjects.

Variable	Statistical	*p*
Gender (%)	*χ*^2^ = 13.124	<0.001
Age (years)	−13.663	<0.001
Glycemic status (%)	*χ*^2^ = 99.696	<0.001
MI (%)	*χ*^2^ = 4.952	0.026
CHD (%)	*χ*^2^ = 5.829	0.016
Hypertension (%)	*χ*^2^ = 28.536	<0.001
Family History of Diabetes (%)	*χ*^2^ = 6.359	0.012
Smoking (%)	*χ*^2^ = 3.663	0.056
Alcohol consumption (%)	*χ*^2^ = 1.766	0.184
Height (cm)	−0.741	0.459
Weight (Kg)	−3.540	<0.001
BMI (Kg/m^2^)	−4.281	<0.001
Pulse (beats per minute)	−2.332	0.020
ALT (U/L)	−2.243	0.025
AST (U/L)	−2.360	0.018
GGT (U/L)	−1.164	0.244
Crea (umol/L)	−4.001	<0.001
TC (mmol/l)	1.873	0.061
TG (mmol/l)	−2.532	0.011
HDL-C (mmol/l)	4.851	<0.001
LDL-C (mmol/l)	0.735	0.463
TyG	−4.876	<0.001
TyG-BMI	−5.105	<0.001

### Association of TyG-BMI quartiles with the risk of IS

**Figure 2:**
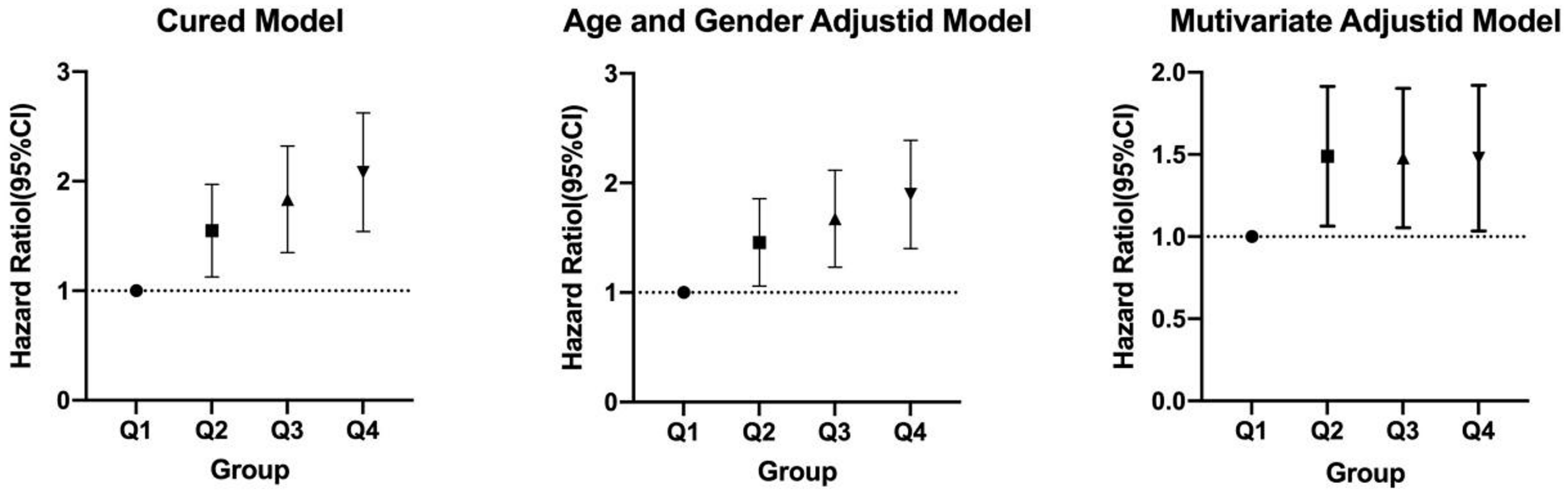
Cox proportional hazards regression analysis of TyG-BMI and the risk of incident ischemic stroke.

As shown in Table 4 and Figure 2, TyG-BMI was categorized into quartiles and analyzed using the Cox proportional hazards regression model. In Model 1 (unadjusted), individuals in the Q2 group had a 51% increased risk of developing new-onset ischemic stroke (IS) within 10 years compared to those in the Q1 group (HR = 1.51, *p* = 0.003). Similarly, the Q3 and Q4 groups had 79.2% (HR = 1.792, *p* < 0.001) and 103.5% (HR = 2.035, *p* < 0.001) increased risks, respectively. In Model 2, after adjusting for age and sex, the risks remained elevated: 42.1% for the Q2 group (HR = 1.421, *p* = 0.013), 63.4% for the Q3 group (HR = 1.634, *p* < 0.001), and 85.2% for the Q4 group (HR = 1.852, *p* < 0.001). In Model 3, which further adjusted for glucose metabolism status and hypertension, the risk remained significantly higher: 44.9% in the Q2 group (HR = 1.449, *p* = 0.012), 43.8% in the Q3 group (HR = 1.438, *p* = 0.014), and 43.4% in the Q4 group (HR = 1.434, *p* = 0.020), relative to the Q1 group.

**Table 4:** Cox proportional hazard regression model for the association between TyG-BMI and the risk of new onset ischemic stroke.

Group	Model 1		Model 2		Model 3	
	HR (95%CI)	*p*	HR (95%CI)	*p*	HR (95%CI)	*p*
Q1	Ref.		Ref.		Ref.	
Q2	1.510 (1.146,1.990)	0.003	1.421 (1.078,1.873)	0.013	1.449 (1.086,1.932)	0.012
Q3	1.792 (1.372,2.340)	<0.001	1.634 (1.251,2.134)	<0.001	1.438 (1.076,1.921)	0.014
Q4	2.035 (1.566,2.645)	<0.001	1.852 (1.425,2.408)	<0.001	1.434 (1.058,1.942)	0.020

Model 1: There are no covariates were adjusted. Model 2: Age and gender were adjusted. Model 3: Age, gender, Glycemic status, hypertension, family history of diabetes, myocardial infarction, Coronary heart disease, pulse, HDL-C, ALT, AST, Crea were adjusted.

### The predictive value of TyG-BMI for the 10-year incidence of new-onset IS

**Figure 3:**
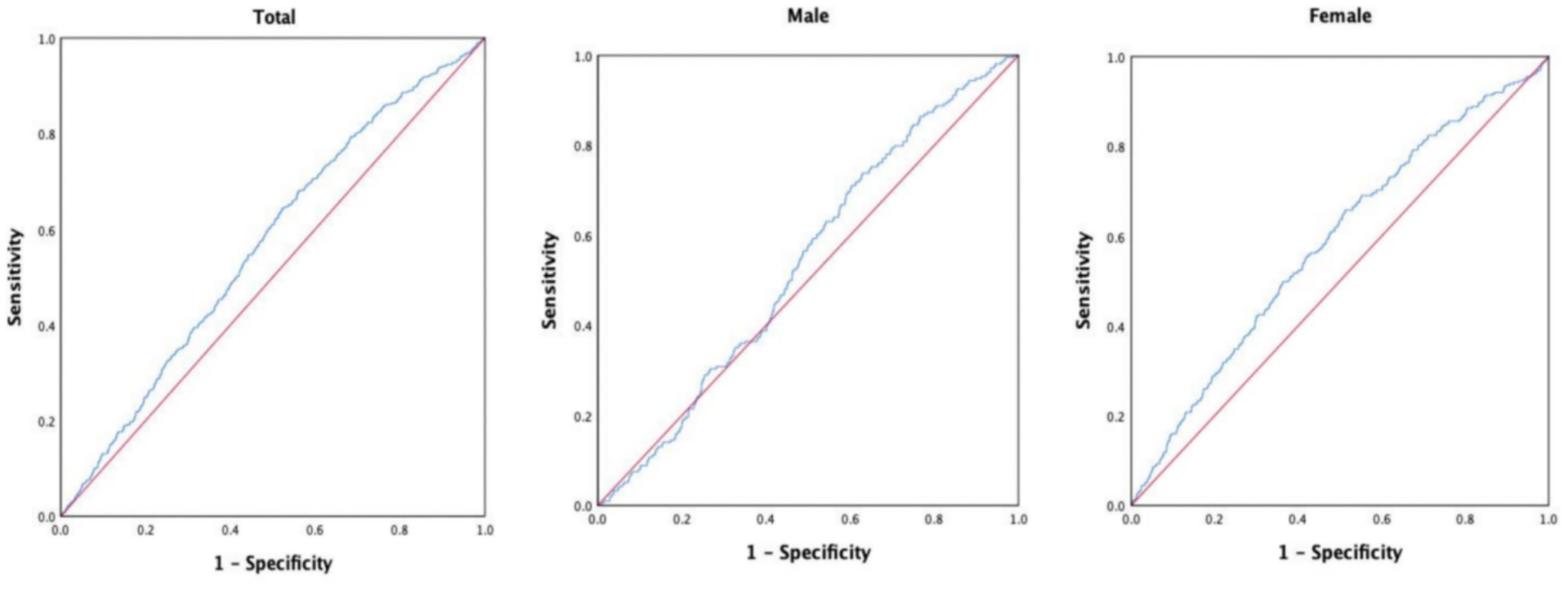
Area under the ROC curve for predicting the occurrence of ischemic stroke.

To explore the predictive value of TyG-BMI for IS, we analyzed the ROC curves (Figure 3). The results showed that, in the entire study population, TyG-BMI predicted the 10-year incidence of new-onset IS with an AUC of 0.566 (95% CI = 0.542–0.590, *p* < 0.001), a cutoff value of 204.1307, a sensitivity of 64.3%, and a specificity of 47.8%. Among male participants, TyG-BMI predicted the 10-year incidence of new-onset IS with an AUC of 0.537 (95% CI = 0.501–0.574, *p* = 0.067), a cutoff value of 195.1996, a sensitivity of 73.8%, and a specificity of 37.0%. In female participants, TyG-BMI predicted the 10-year incidence of new-onset IS with an AUC of 0.583 (95% CI = 0.551–0.615, *p* < 0.001), a cutoff value of 204.295, a sensitivity of 65.8%, and a specificity of 48.7%.

## Discussion

This study is based on epidemiological research methods and established a non-interventional prospective cohort study. Through a follow-up period of up to 10 years, we investigated the incidence of IS among the participants. We aimed to explore the predictive value of TyG-BMI for the 10-year incidence of new-onset IS in the middle-aged and elderly population of Luzhou City. When examining disease risk factors, non-interventional prospective cohort studies offer clear advantages over cross-sectional studies. Through both univariate and multivariate analyses, we assessed the correlation between TyG-BMI and the risk of new-onset IS in the elderly population of Luzhou City with over 10 years of follow-up. Specifically, the correlation between TyG-BMI and new-onset IS was found to be strong, with the cumulative incidence of IS significantly increasing across TyG-BMI quartiles. Both univariate and multivariate analyses revealed a significant correlation between TyG-BMI and new-onset IS. ROC curve analysis demonstrated that the cutoff value of TyG-BMI for predicting 10-year new-onset IS in the entire study population was 204.1307 long-term predictor of IS in middle-aged and older adults, adding to the growing body of literature on insulin resistance-related indices and cerebrovascular risk. Previous cohort studies, such as one based on data from the UK Biobank, have demonstrated a progressive increase in stroke risk across ascending quartiles of TyG, TyG-BMI, and TyG-WC, reinforcing the potential value of these indices in stratifying cardiovascular and cerebrovascular risk over time (16). Furthermore, cross-sectional evidence from the China Health and Retirement Longitudinal Study (CHARLS) also supports this association. That study revealed that both TyG and TyG-BMI were significantly associated with elevated stroke risk, with adjusted odds ratios (ORs) ranging from 1.186 to 1.246 and consistent 95% confidence intervals, suggesting a robust link even after adjustment for multiple confounders (17). In addition to general population data, the predictive value of TyG-BMI has been corroborated in critically ill patients. A study utilizing the eICU database found that individuals in the high TyG-BMI group experienced significantly higher 28-day hospital and ICU mortality following ischemic stroke (HR = 1.734, *p* = 0.032 for hospital mortality; HR = 2.337, *p* = 0.048 for ICU mortality), highlighting the prognostic relevance of TyG-BMI even in acute clinical settings (18). Together, these findings support the consistency and generalizability of our results across different populations and study designs. Our study extends the current knowledge by providing prospective, 10-year follow-up data from a large Chinese cohort, further substantiating the TyG-BMI index as a simple yet powerful tool for long-term stroke risk prediction. Future research should continue to explore the biological mechanisms underlying these associations and assess whether TyG-BMI can serve as a modifiable risk marker in clinical practice.

IR is characterized by a reduced sensitivity to insulin, leading to compensatory increases in insulin secretion (19). This metabolic disorder affects the cerebrovascular system through various mechanisms, thereby increasing the risk of IS (19). Under normal conditions, insulin promotes vasodilation by stimulating the synthesis of nitric oxide (NO) (20). However, in the IR state, this signaling pathway is impaired, resulting in reduced bioavailability of NO, which in turn leads to vasoconstriction, inflammatory responses, and thrombosis. Furthermore, IR can upregulate the expression of cell adhesion molecules, facilitating the adhesion and migration of white blood cells, thus further accelerating the development of (21, 22). In individuals with IR, elevated levels of plasminogen activator inhibitor-1 (PAI-1) inhibit fibrinolysis, increasing the likelihood of (23, 24). Additionally, IR promotes platelet aggregation, further enhancing the risk of arterial occlusion (25). IR is often accompanied by increased levels of TG, low HDL-C, and small, dense low-density lipoprotein (LDL) particles (26), all of which contribute to the accelerated formation of atherosclerotic plaques and elevate the risk of cerebrovascular events.

The TyG index, calculated from fasting TG and blood glucose, serves as a reliable alternative for assessing insulin resistance. BMI reflects an individual’s body fat level, and when combined with the TyG index to form TyG-BMI, it provides a more comprehensive assessment of metabolic risk. Studies have shown that TyG-BMI more accurately reflects insulin resistance than either the TyG index or BMI alone and is closely associated with the risk of IS. A study targeting middle-aged and elderly populations found that higher TyG-BMI levels were significantly associated with an increased incidence of stroke, with this association remaining significant even after adjusting for traditional risk factors (27). Research has shown that patients with acute ISand higher TyG-BMI levels may have better short-term prognoses than those with lower TyG-BMI levels, potentially due to the “obesity paradox, “where moderate overweight may provide metabolic reserves that improve post-stroke recovery (28).

TyG-BMI is strongly associated with an increased risk of IS. The underlying biological mechanisms may involve IR, which contributes to endothelial dysfunction and dyslipidemia. These alterations promote the formation of plaques in the carotid and cerebral arteries and increase plaque instability, ultimately leading to ischemic events. In addition, IR may impair cerebral autoregulation, reducing the brain’s ability to maintain stable perfusion during fluctuations in blood flow, thereby elevating the risk of cerebral ischemia. IR is also closely linked to chronic low-grade inflammation. Elevated levels of inflammatory mediators, such as TNF-*α* and IL-6, further damage endothelial function and accelerate the progression of atherosclerosis (29).

From a clinical perspective, these findings suggest that TyG-BMI should be incorporated into the risk assessment system for ISto more effectively identify high-risk populations. For individuals with higher TyG-BMI, measures to improve insulin sensitivity, such as healthy eating, regular exercise, weight loss, and pharmacological interventions, may be key strategies to reduce stroke incidence.

This study found that elevated TyG-BMI levels are closely associated with an increased risk of IS over the next 10 years. This finding further supports the critical role of IR in the onset and progression of IS. By examining the relationship between IR, metabolic disturbances, and cerebrovascular events, the study underscores the importance of incorporating TyG-BMI into comprehensive stroke risk assessments and prevention strategies.

### Limitations

This study has several limitations that should be acknowledged. First, the identification of IS events was based on data from the chronic disease management system of the Health Commission and the Center for Disease Control, which may have led to underreporting or misclassification in a small number of cases. Future investigations should consider incorporating in-person follow-up assessments, where feasible, to enhance the completeness and accuracy of outcome data. Second, the extended follow-up period of 10 years introduces potential confounding factors, including the development of comorbidities, initiation or modification of pharmacological treatments, and substantial lifestyle changes over time. While these factors are largely unavoidable in long-term observational studies, they may have influenced the observed associations. Nonetheless, such real-world variability reflects clinical practice and does not substantially detract from the generalizability or interpretability of the findings.

## Conclusion

This study revealed a significant association between TyG-BMI and the 10-year incidence of new-onset IS among middle-aged and elderly individuals, indicating that TyG-BMI may serve as a valuable predictive marker for assessing IS risk in this population.

## Data Availability

The raw data supporting the conclusions of this article will be made available by the authors, without undue reservation.
